# Gentamicin concentration and acute kidney injury in term neonates treated for neonatal sepsis with gentamicin and ampicillin-cloxacillin combination

**DOI:** 10.1128/aac.01495-23

**Published:** 2024-05-15

**Authors:** Masanyiwa Ernest James, Helga Naburi, Sabina Mugusi, Peter P. Kunambi, Tosi Mwakyandile, Rajabu Hussein Mnkugwe, Mohamed Ally Khalfan, Mainen J. Moshi, Omary M. Minzi, Philip Sasi

**Affiliations:** 1 Department of Clinical Pharmacology, School of Biomedical Sciences, Campus College of Medicine, Muhimbili University of Health and Allied Sciences, Dar es Salaam, Tanzania; 2 Department of Medical Physiology, School of Medicine and Dentistry, The University of Dodoma, Dodoma, Tanzania; 3 Department of Paediatrics and Child Health, School of Clinical Medicine, Campus College of Medicine, Muhimbili University of Health and Allied Sciences, Dar es Salaam, Tanzania; 4 Department of Biological and Pre-clinical Studies, Institute of Traditional Medicine, Muhimbili University of Health and Allied Sciences, Dar es Salaam, Tanzania; 5 Department of Clinical Pharmacy and Pharmacology, School of Pharmacy, Muhimbili University of Health and Allied Sciences, Dar es Salaam, Tanzania; Columbia University Irving Medical Center, New York, New York, USA

**Keywords:** population pharmacokinetics, neonatal sepsis, acute kidney injury, therapeutic drug monitoring, gentamicin

## Abstract

Gentamicin is widely used to treat neonatal infections caused by both Gram-negative and Gram-positive bacteria, and the WHO recommends its use while monitoring serum creatinine and gentamicin concentrations to avoid drug-induced nephrotoxicity and ototoxicity. Yet in some resource-limited settings, the drug is used without monitoring. A population pharmacokinetics study involving term neonates with neonatal infection admitted to a neonatal unit. Participants were started on intravenous gentamicin 5 mg/kg once a day in combination with ampicilin-cloxacillin. Blood samples for serum gentamicin concentration were taken at 0.25, 0.5, 1, 2, 3, 5, 6, 8, 10, 12, 14, 16, 18, 20, 23, and 24 hours after the initial dose, each participant contributing two samples to the 24 hour sampling schedule. An additional sample for trough concentration was taken from each participant just before the third gentamicin dose while serum creatinine concentration was measured before and after treatment. Twenty-four participants were enrolled into the study and included in the final analysis. Mean (SD) peak and trough serum gentamicin concentrations were 16.66 (0.64) µg/mL and 3.28 (0.70) µg/mL, respectively. Gentamicin clearance (CL) was 0.40 mL min^−1^ kg^−1^ and volume of distribution (VD) was 0.31 L kg^−1^. Mean (SD) serum creatinine level after treatment was 209.7 (70.4) µmol/L compared to 103.3 (23.6) µmol/L before treatment [mean difference (106.4 ± 67.1; 95% confidence interval (CI): 78.1; 134.7 µmol/L; t (23) = 7.77; *P* < 0.001]. All participants fulfilled the Kidney Disease Improving Global Outcomes (KDIGO) criteria for acute kidney injury after treatment. Treatment of neonatal infection with antimicrobial regimen containing gentamicin, without renal function and gentamicin concentration monitoring, carries a significant risk for drug-induced acute kidney injury.

## INTRODUCTION

Children face the highest risk of dying in their first month of life, making this period crucial for survival to the fifth birthday. Pre-term birth, intrapartum-related complications, infections, and birth defects are the leading associates of this high risk. Despite reduction by 52%, from 38 deaths per 1,000 in the period between 1990 and 2019 to an average global rate of 17 neonatal deaths per 1,000 live births reported in 2021, neonatal death rate remains unacceptably high (1). Neonatal sepsis, a form of infection, accounts for 30–50% of these deaths each year and is considered to be among the commonest causes of neonatal morbidity and mortality worldwide (2). In Tanzania, where current neonatal mortality is 20.3 deaths per 1,000 live births, neonatal sepsis accounts for 29% of these deaths (3). Therefore, effective treatment of early-onset sepsis may significantly contribute to reduction of neonatal mortality.

Treatment of neonatal sepsis is usually initiated with empirical dosing using antibiotics that cover both Gram-positive and Gram-negative bacteria. In Tanzania, the standard treatment guideline specifically recommends a combination of gentamicin and ampiclox (a combination of ampicillin and cloxacillin) as the first-line antibiotic combination (4). In this combination, gentamicin (5 mg/kg) is administered as a once-daily bolus injection and ampiclox (100 mg/kg) as a twice-daily bolus injection for 7–10 days (5) or until blood culture results become available. However, gentamicin exhibits a narrow therapeutic index and is known to be nephrotoxic and ototoxic particularly when given for more than 48 hours without dose adjustment guided by serum creatinine and serum gentamicin levels (6). Indeed, up to 25% of all patients who receive gentamicin therapy for more than 48 hours without monitoring of gentamicin concentration and renal function will develop acute kidney injury (AKI) that may progress to chronic kidney disease (CKD) (7). Consequently, in neonates, the WHO recommends therapeutic drug monitoring (TDM) and kidney function testing before, during, and after treatment with gentamicin (6). In Tanzania, where this study was conducted, TDM and renal function monitoring are not routinely done for various reasons including lack of equipment and high cost of performing blood tests as this service is not covered by the National Health Insurance Fund. This raises a concern that the number of neonates who may be suffering from AKI following gentamicin exposure and who might later develop CKD and/or hearing loss may be high. We conducted a sparse sampling population pharmacokinetics (PopPK) study to determine the serum gentamicin concentration following initial dose and the day-two trough concentration among term neonates admitted to a neonatal unit and treated with intravenous gentamicin, 5 mg per kilogram per day for 5–7 days, in order to evaluate the serum levels achieved and their effect on renal function

## MATERIALS AND METHODS

### Study design, period, and setting

This was a population pharmacokinetics (PopPK) study where a limited number of blood samples (sparse sampling) for gentamicin concentration were drawn from each participant results of which were used to construct a population pharmacokinetic profile following the initial dose. The study was conducted between May and June 2021 in a neonatal unit at Mwananyamala Regional Referral Hospital (MRRH). This is a designated regional referral hospital located in Dar es Salaam that receives patients from different parts of the city. The neonatal unit at MRRH is the second biggest neonatal unit after the one at Muhimbili National Hospital and it admits nearly 400 neonates per month. The unit has adequate number of medical doctors and nurses including experienced pediatricians. Neonatal care services are supported with a well-equipped laboratory with the capacity to perform biochemical tests including serum creatinine.

### Study population and eligibility criteria

This study involved term neonates (born at gestational age >37 completed weeks) admitted at MRRH during the study period. The inclusion criteria were aged 1–28 days, prescription of gentamicin and ampiclox intended for treatment for longer than 48 hours, and written consent to participate in the study given by the mother. Exclusion criteria were severe illness in decompensated state (respiratory distress, lethargy, comatose state, impaired tissue perfusion, and any other condition for which the attending physician decided that resuscitation was indicated) requiring resuscitation, severe congenital malformation such as anencephaly, use of other medications that have potential nephrotoxicity or ototoxicity (vancomycin, furosemide, acyclovir, amphotericin B, and ibuprofen) within the past 72 hours before enrolment, or use of other medications that have potential pharmacokinetic interaction with gentamicin (phenobarbital, erythromycin, and phenytoin) within the past 72 hours before enrolment and being on gentamicin therapy within the past 72 hours. Eligible patients (meeting all the inclusion criteria and none of the exclusion criteria) were consecutively enrolled.

### Sample size estimation

The sample size was estimated according to standard pharmacokinetic modeling and simulation using the confidence interval approach (8). Setting the estimation of the 95% CI for clearance and volume of distribution (main parameters estimated from the PopPK profile) at 20% precision and power of 80%, the required sample size was calculated to be 20–24 participants (9).

### Data collection procedures

Data were collected using a specially designed case report form. Information recorded included demographic information (age, gender, birth weight, height, and gestational age), Apgar score, clinical diagnosis, gentamicin dosing, and blood sampling (serum creatinine and gentamicin pharmacokinetics).

### Study procedures

Mothers were approached upon baby's admission to the neonatal unit, and invited for their neonate to participate in the study. Neonates of consenting mothers were then screened for eligibility and those eligible were recruited. Study participation involved collection of blood samples for serum creatinine on day 0 and day 7 (from start of gentamicin treatment) and gentamicin pharmacokinetics following the initial gentamicin dose on day 0 using a sampling schedule determined prior to start of the study. Additionally, a blood sample for gentamicin day-two trough concentration was taken from each participant on day 2 from start of treatment.

### Drug administration, blood sampling, and determination of serum gentamicin concentration

Gentamicin (5 mg/kg) was administered once-daily and ampiclox (100 mg/kg) twice-daily where both medications were administered as intravenous bolus injections.

A blood sample for population PopPK, 0.5 mL, was drawn via a heel prick from each participant at different scheduled sampling times within 24 hours after administration of the first dose of gentamicin. Samples were taken at 0.25, 0.5, 1, 2, 3, 5, 6, 8, 10, 12, 14, 16, 18, 20, 23, and 24 hours post dosing. Each participant contributed two samples**,** at different time points, to the 24 hour sampling schedule. The sampling schedule for all participants was designed to cover the entire range of serum gentamicin concentrations that would make the concentration versus time profile from extensive sampling. This profile was used to estimate gentamicin PK parameters (CL and VD) for this population. Samples for day-two serum gentamicin trough concentration were drawn from each participant on day 2, 5 minutes before the third gentamicin dose.

Blood samples for both PopPK and trough concentration were collected in red topped serum separator tubes (with granules) and were immediately stored at −20°C before being transported in a cool ice box (2–8°C) to central pathology laboratory (CPL) at Muhimbili National Hospital within 1 hour of collection. At CPL, serum was transferred to another tube and centrifuged at 3,000 relative centrifugal force for 10 minutes and stored at −80°C before analysis for gentamicin concentration using chemiluminescent microparticle immunoassay technology (Abbott architect ci4100 analyzer; Abbott diagnostics in Illinois, United States), at the end of the study.

### Determination of serum creatinine concentration and criteria for acute kidney injury

Blood samples (0.5 mL, heel prick) for serum creatinine (SCr) were collected into red top serum separator tubes (with granules), from all participants on days 0 and 7. After collection, these samples were immediately taken to the MRRH laboratory for processing where they were left on the bench for serum to separate before being transferred into pink top tubes, centrifuged, and then analyzed (fully automated Erba XL-100 analyzer; Erba Mannheim Diagnostics Company, India).

In this study, high creatinine concentration was defined as a serum creatinine level above 132 µmol/L (1.5 mg/dL). In term neonates, physiological immaturity of the kidneys, dehydration, birth asphyxia, sepsis, maternal factors such as preeclampsia, genetic factors affecting kidney development, and medications such as gentamicin may predispose neonates to high creatinine concentrations. Gentamicin exposure is a well-defined cause of nephrotoxicity with clear mechanism of action. Unlike elevated serum creatinine concentration from other causes, gentamicin-induced creatinine elevation typically occurs early during treatment and is often reversible upon discontinuation of the medication.

Acute kidney injury was defined using the Kidney Disease Improving Global Outcomes (KDIGO) criteria in neonates that defines AKI as an increase in serum creatinine by 0.3 mg/dL or more within 48 hours or increase in serum creatinine to 1.5 times baseline or more within the last 7 days (10). The increase to 1.5 times or more within 7 days was the definition used in this study.

### Data analysis

Data were entered and analyzed using (StataCorp. 2019. Stata Statistical Software: Release 14.2. College Station, TX: StataCorp LLC). Assuming a one compartment model and first order kinetics, the serum gentamicin concentrations versus time profile was fitted to give the characteristic serum concentration time curve following an intravenous dose. From this curve, PopPK parameters were directly estimated (elimination rate constant, elimination half-life, and area under the curve). Using these PopPK parameters, other important PopPK parameters (CL and VD) were calculated.

The Kolmogorov-Smirnov and Shapiro-Wilk tests were used to determine the normality of data distribution, and our data were found to be normally distributed. Continuous data were expressed as mean ± SD (standard deviation) and were compared using one sample *t*-test (comparison of the observed mean day-two trough serum gentamicin concentration and the upper limit of the reference range) and paired samples *t*-test (comparison of the mean serum creatinine concentration before and after treatment with gentamicin). Proportions of neonates with high serum creatine concentration before and after gentamicin treatment were compared using MacNemar test. The results were considered statistically significant at *P < 0.05*.

## RESULTS

**Fig 1:**
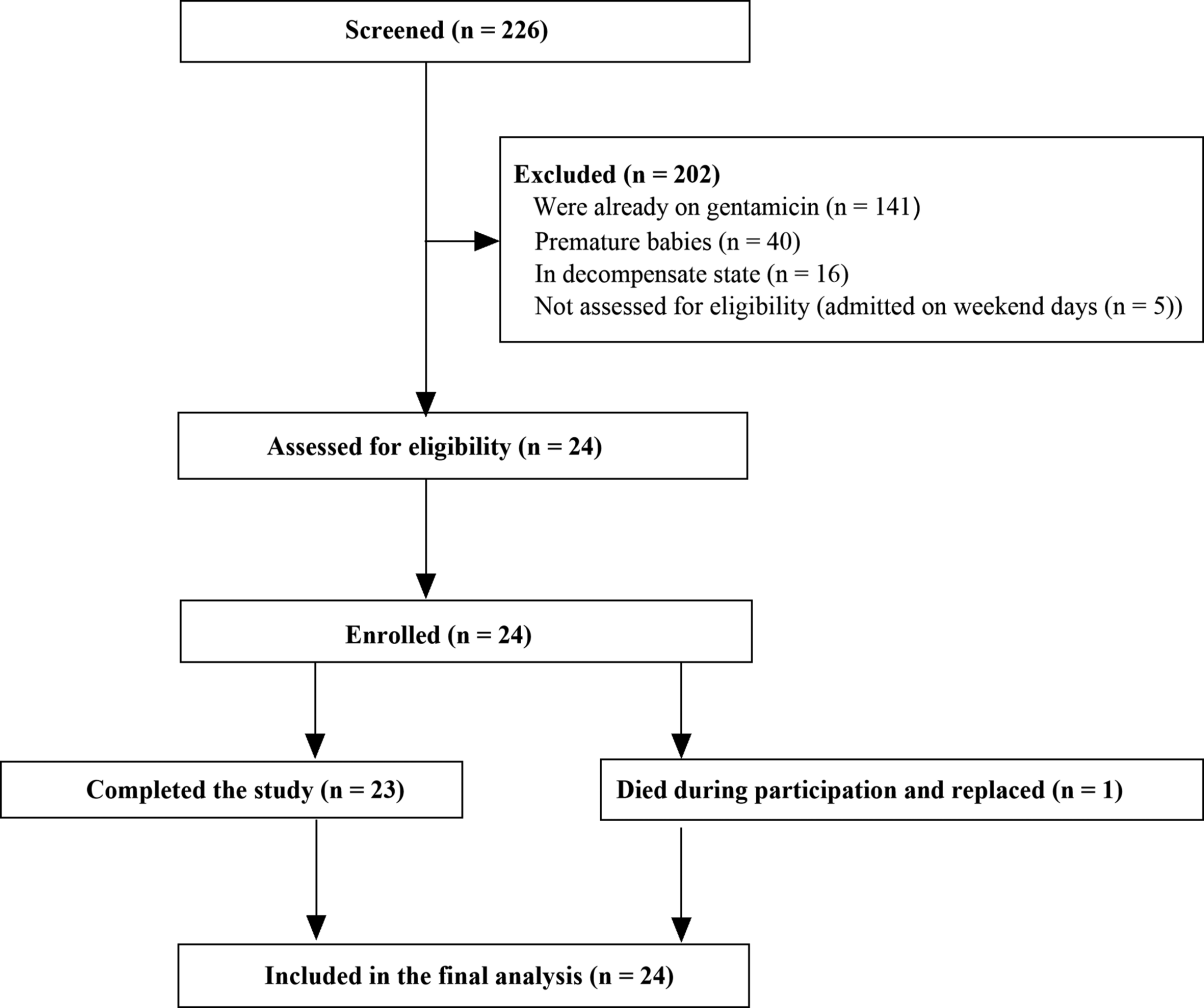
Patient recruitment and participation in the study.

A total of 226 neonates were screened and 202 of these were screen failures for various reasons (Fig. 1). Prior gentamicin exposure, was the commonest reason (62.6%) for screen failure. The remaining 24 were assessed for eligibility; all were eligible, and their mothers consented and were enrolled into the study.

### Social demographic and clinical data

The majority of study participants were male (56%). The median gestational age (GA) of the study population was 38 weeks (range 37, 40), which corresponded with their mean birth weight of 3.12 (SD ± 0.61) kg. The mean Apgar score was 8.0 (SD ± 0.2) (Table 1), and most of the study participants (72%) had clinical diagnosis of neonatal sepsis.

**TABLE 1:** Social demographic and clinical characteristics of the study participants (*N* = 25)

Characteristics	Frequency	%
Age group (days)		
0–7	18	75
8–15	2	8.3
16–28	4	16.7
Sex		
Male	14	56.0
Female	11	44.0
Mean birth weight in kg (±SD)	3.12 ± 0.61	
Mean height in cm (±SD)	50.16 ± 3.03	
Median gestational age in weeks (range)	38 (37, 40)	
Mean Apgar score (±SD)	8.0 ± 0.2	
Clinical diagnosis		
Neonatal sepsis	18	72.0
Neonatal jaundice	3	12.0
Meconium aspiration	1	4.0
Pneumonia	1	4.0
Birth asphyxia	2	8.0
Other clinical records		
Diarrhea	5	20
Unable to suck	3	12

### Serum trough gentamicin levels

All 24 participants contributed a blood sample for day-two trough serum gentamicin concentration. All 24 participants had higher day-two trough serum gentamicin levels (>2 µg/mL) than the reported upper limit of normal for a typical term neonate (≤2 µg/mL). Mean day-two trough concentration (SD) was 3.28 (±0.70); 95% CI = 2.98; 3.58 µg/mL; t (23) = 8.893; *P* < 0.001, one sample *t*-test using reported upper limit of normal as the null value (Ho = 2 µg/mL).

### Population pharmacokinetic parameters

**Fig 2:**
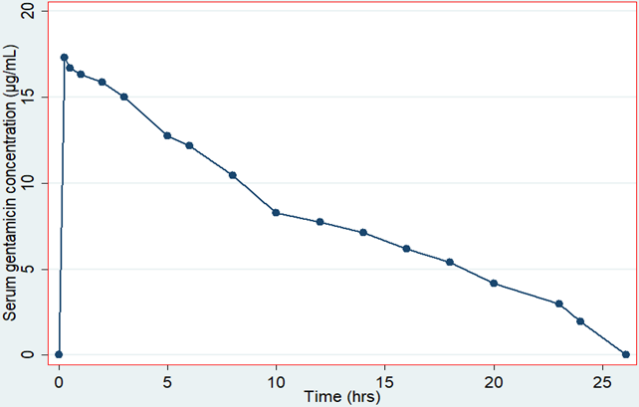
Population serum gentamicin concentration (µg/mL) versus time profile for participating neonates.

For the PopPK profile, a total of 45 serum samples were analyzed for serum gentamicin concentration. The averages of serum gentamicin concentrations at each time point were used to plot the population serum gentamicin concentration versus time profile (Fig. 2). Assuming one compartment model, the profile was fitted using stata software, StataCorp. 2019. Stata Statistical Software: Release 14.2. College Station, TX: StataCorp LLC (Fig. 2). PopPK parameters were directly estimated from the curve where the elimination rate constant was 0.0793 h^−1^; elimination half-life (t½) was 8.7 hours, and area under the curve was 206.11 µg mL^−1^ h^−1^. Using these popPK parameters, CL and VD were calculated and were 0.40 mL min^−1^ kg^−1^ and 0.31 L kg^−1^, respectively.

### Change in serum creatinine concentration after gentamicin treatment

**Fig 3:**
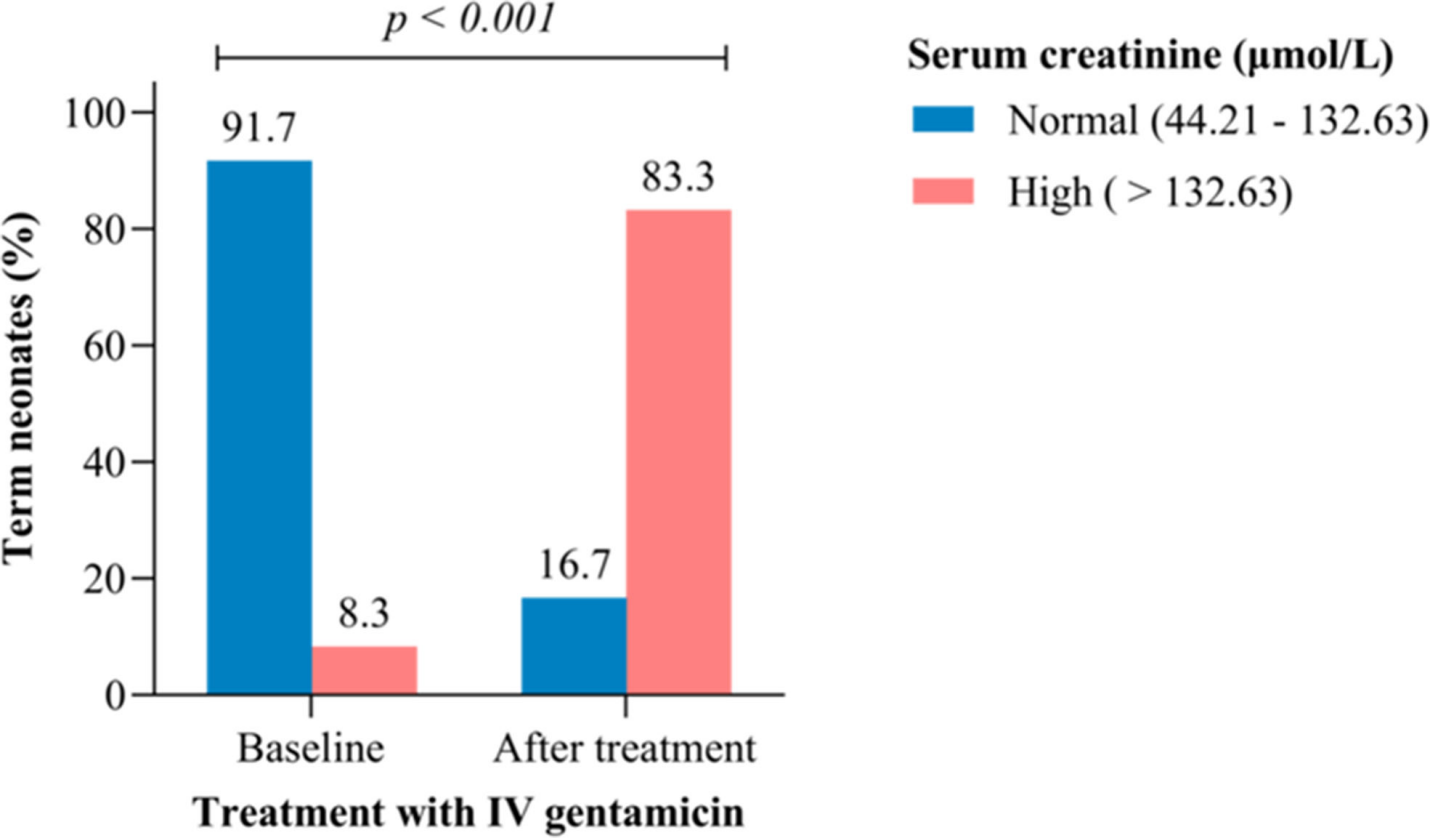
Percentage of high serum creatinine levels before and after treatment with gentamicin.

A total of 24 paired serum samples (before and after treatment with gentamicin) from each participant were analyzed for serum creatinine level. Before treatment, 2 (8.3%) participants had high serum creatinine level and the number with high serum creatinine level increased to 20 (83.3%) participants after treatment (Fig. 3). This shows a 10-fold increase in the number of participants with high serum creatinine levels (>132 µmol/L), 7 days from start of gentamicin treatment. The mean (SD) serum creatinine level after treatment with gentamicin, 209.7 (70.4) µmol/L, was significantly higher than the mean (SD) serum creatinine level before treatment, 103.3 (23.6) µmol/L (paired sample *t*-test) mean difference [106.4 ± 67.1; 95% CI: 78.1; 134.7 µmol/L; t (23) = 7.77; *P* < 0.001], assuming no difference in mean creatinine concentration before and after treatment (Ho = 0).

### Acute kidney injury

Fourteen (58.3%) of the study participants had baseline serum creatinine levels increased by a factor of 1.5–2 after treatment and had suffered AKI stage I (based on updated KDIGO criteria) while 10 (41.67%) of the study participants had baseline serum creatinine levels increased by a factor of 2–3 and had suffered AKI stage II. Therefore, all participants in our study had met the criteria for acute kidney injury after being treated with gentamicin.

## DISCUSSION

In our study, we show that day-two trough serum gentamicin concentrations in term neonates, after administration of the recommended dose in the absence of dose adjustment and renal function monitoring, exceed the toxic concentrations of >2 µg/mL. These data lend support for the recommendation to perform routine serum creatinine test before, during, and after treatment with gentamicin, and to monitor serum gentamicin levels during treatment (11). Different studies on clinical pharmacokinetics of gentamicin in neonates including a study by Pacifici et al*.* show that neonates who achieve steady state peak serum gentamicin levels above 12 µg/mL and trough serum gentamicin levels above 2 µg/mL develop AKI (6, 7, 11, 12). Our findings are consistent with other studies conducted recently including another study by Pacifici et al*.* on gentamicin-induced nephrotoxicity in neonates, which shows that, in circumstances of no baseline serum creatinine monitoring, no regular renal function assessment during treatment, and no gentamicin level monitoring, patients achieve higher trough serum gentamicin levels that increase the risk of nephrotoxicity (12). This raises safety concerns when gentamicin is used without monitoring and highlights the need for serum creatinine levels and gentamicin concentration monitoring during treatment. The normal and safe practice requires that baseline creatinine concentrations be checked for all patients and the dose to be given, the frequency of dosing, and timing of the next dose should depend on the patient's renal function (12). Therefore, there is a need to seriously consider implementing monitoring of baseline serum creatinine and measuring gentamicin concentrations in neonates admitted to health facilities in resource-limited settings including Tanzania where gentamicin TDM and serum creatinine monitoring are not part of standard of care and yet the aminoglycoside remains the first-line antibiotic for empirical treatment of neonatal sepsis. This will ensure safety as the doses are calculated according to the patient's renal function and serum gentamicin concentration thereby preventing trough concentrations reaching the toxic range.

We also demonstrate that, in term neonates treated for sepsis, gentamicin clearance (CL) and volume of distribution (VD) are lower than the reported reference ranges. Gentamicin CL was 0.40 mL min^−1^ kg^−1^ and VD was 0.31 L kg^−1^ whereas the normal reference ranges are 0.50–1.71 mL min^−1^ kg^−1^ for CL (12) and 0.4–0.7 L kg^−1^ for the VD (13). These findings show that the peak concentration following the initial dose correlates with the day-two trough concentration, both being higher than the upper limit of reference range. This possibly implies that the higher serum gentamicin levels observed among the study participants indicate lower gentamicin CL and or VD. Sepsis, impaired renal function, and birth asphyxia observed in neonates in different studies are thought to lower gentamicin renal CL (6, 14). In our study, majority of the study participants (72%) had clinical diagnosis of sepsis, 8.3% had impaired renal function before starting gentamicin treatment, and 8.3% had clinical diagnosis of birth asphyxia; thus, these clinical conditions may have contributed to the observed lower gentamicin CL. This further highlights the need for baseline assessment of renal function before initiating gentamicin and monitoring during treatment in neonates as they are likely to have impaired renal function although the numbers with impaired renal function and asphyxia before gentamicin exposure, in this study, are small. Neonates have a larger extracellular fluid volume (ECF) and therefore highly water-soluble drugs that are distributed predominantly in the ECF such as gentamicin are expected to have large VD. The large amount of extracellular body water in neonates will tend to result in lower serum gentamicin concentrations. This is contrary to the findings of our study where serum gentamicin levels were higher and the VD was lower. We speculate that lower ECF volume resulting from inadequate fluid or fluid loss among our study participants may partly explain the low VD. It has been shown that, any factor, which reduce the ECF volume tend to decrease the VD of gentamicin (15). In our study, 20% of the study participants had diarrhea and 12% were unable to suck and were put on IV fluids (dextrose-normal saline). Diarrhea and inadequate feeds in neonates tend to reduce the ECF and may lead to reduced VD and gentamicin CL. Findings from a study by Pacifici et al*.* highlighted the importance of closely monitoring fluid balance and correcting dehydration prior commencing treatment with gentamicin (12). This ensures that the extracellular fluid volume is kept normal thereby attaining serum gentamicin levels within the accepted range to prevent the risk of nephrotoxicity.

In the absence of serum gentamicin concentration monitoring, we have observed a significant increase in serum creatinine levels indicating gentamicin-induced nephrotoxicity. The mean serum creatinine level after treatment was higher by 106.4 µmol/L compared to the mean serum creatinine level before treatment. Nephrotoxicity is considered significant when there is mean increase in serum creatinine levels of 44.21 µmol/L and above from the baseline levels (16). In this study, the mean increase in serum creatinine levels was three times this minimum level for nephrotoxicity and can be suggested that significant renal injury occurred in the study participants as the result of gentamicin accumulation. It was observed that 8.3% of the study participants had high serum creatinine levels at baseline and this number increased 10-fold (83%) within 7 days from start of gentamicin treatment. This implies the real potential for gentamicin-induced nephrotoxicity in neonates put on treatment and justifies the need for renal function and serum gentamicin concentration monitoring. However, our study was non-comparative and other causes of creatinine increase in term neonates cannot be ruled out completely.

Neonates are known to have greatly elevated serum creatinine concentration in relation to size and muscle mass and remain elevated up to two weeks from birth (17). The levels we observed in our study were even higher than expected for term neonates.

The traditional indicator of AKI is a rise in serum creatinine concentration, which forms the basis of all current AKI definitions. In our study, based on the updated KDIGO criteria (18), about 58% of the study participants developed AKI stage I whereas about 42% developed AKI stage II. In this study, therefore, all study participants developed AKI after treatment with gentamicin. In comparison with other studies on gentamicin-associated AKI, our study had a large proportion of study participants who developed AKI. For example, in a study by Selby et al*.* on gentamicin-associated acute kidney injury on patients treated with gentamicin, AKI occurred in 24.4% patients (19). This difference with our finding may be contributed by the difference in trough serum gentamicin levels achieved by the study participants. In Selby et al*.* study, the overall steady state trough serum gentamicin concentration was 2.37 (±0.24) µg/mL while in our study, it was 3.283 (±0.707) µg/mL, this difference in trough serum gentamicin concentration can explain the observed difference because the higher the trough serum concentration achieved, the more damage to the kidneys occurs (16). Evidence from different studies suggest that, patients who have recovered from AKI have a 25% increased risk for developing progressive CKD and even end-stage renal disease (20).

### Study limitations

This study has several limitations: the overall limitation was on reliance on serum creatinine to categorize AKI; we defined AKI solely based on serum creatinine that may have limitation. Combining serum creatinine level and other biomarkers of kidney injury to define AKI may improve specificity (21). This could be done using urine biomarkers (kidney injury molecule-1 and neutrophil gelatinase-associated lipocalin), which are considered to be more specific and sensitive markers for early prediction of AKI (22, 23). Also, the variation of serum gentamicin concentration among participants (at each time point), which might have influenced the observed results, was not determined. However, we believe that this variation was not significant as most of participants in our study were from similar geographical location, born of black mothers, had minor differences in age and weight, and also majority had similar clinical diagnosis (neonatal sepsis). Another limitation was a reliance **only** on first day blood sampling (within 24 hours after initial dose) than taking the full course of trough and peak blood samples during treatment period of 5–7 days. Another limitation of our study is that it was non-comparative in nature. As such, while gentamicin exposure was most likely the cause of creatinine increase, other factors such as dehydration, birth asphyxia, sepsis, and hemodynamic changes could have contributed to this outcome. Despite the limitation, this study provides a snapshot of a real situation with regard to the safety of treating neonatal infection with gentamicin in resource-limited settings.

All our participants had serum gentamicin level in the toxicity range and all developed AKI. This raises the possibility that the current recommended dose may be high for neonates in some settings and dose evaluation in future studies may be justified.
